# Drug-related catatonia in youths: real-world insights from the WHO Safety Database

**DOI:** 10.1007/s00787-023-02234-4

**Published:** 2023-06-12

**Authors:** Diane Merino, Alexandre O. Gérard, Thibaud Lavrut, Florence Askenazy, Susanne Thümmler, François Montastruc, Milou-Daniel Drici

**Affiliations:** 1 Department of Psychiatry, University Hospital of Nice, Nice, France; 2 Department of Pharmacology and Pharmacovigilance Center of Nice, University Hospital Center of Nice, Nice, France; 3 Department of Child and Adolescent Psychiatry, Children’s Hospitals of Nice, CHU-Lenval Nice, Nice, France; 4 CoBTek Laboratory, Université Côte d’Azur, 06000 Nice, France; 5 Department of Medical and Clinical Pharmacology, Centre of PharmacoVigilance and Pharmacoepidemiology, Faculty of Medicine, Toulouse University Hospital, Toulouse, France

**Keywords:** Catatonia, Child psychiatry, Safety, Pharmacovigilance, Side effect

## Abstract

Catatonia is characterized by psychomotor alterations and reduced contact with the environment. Initially linked to schizophrenia, it also occurs in mood disorders or organic conditions. In children, catatonia remains poorly delineated, despite dramatically increasing the risk of premature death. As data on pediatric drug-induced catatonia bears many uncertainties, we aimed to characterize its age-dependent patterns, using real-world data from the WHO safety database (VigiBase®).VigiBase® was queried for all reports of catatonia registered up to December 8th 2022. Reports involving patients <18 years were classified into 3 groups: ≤23 months, 2–11 years, and 12–17 years. Disproportionality analyses relied on the Reporting Odds Ratio (ROR), and the positivity of the lower end of the 95% confidence interval of the Information Component (IC) was required to suspect a signal. Catatonia was evoked in 421 pediatric reports. In infants, vaccines were leading. In children, the main signals involved haloperidol (ROR 104.3; 95% CI 45.6–238.5), ondansetron (ROR 40.5; 95% CI 16.5–99.5), and ciclosporin (ROR 27.4; 95% CI 13.8–54.1). In adolescents, chlorpromazine (ROR 199.1; 95% CI 134.8–294.1), benzatropine (ROR 193; 95% CI 104.1–361.6), and olanzapine (ROR 135.7; 95% CI 104.6–175.9) reached the highest RORs. In infants, catatonia was related to vaccines, it was ascribed to multiple drugs in children, and mainly to psychotropic drugs in adolescents. Less suspected drugs, such as ondansetron, were highlighted. Despite limitations inherent in spontaneous reporting systems, this study supports that a careful anamnesis is warranted to separate catatonia associated with medical conditions from drug-induced catatonia in pediatric patients.

**Supplementary Information:**

The online version contains supplementary material available at 10.1007/s00787-023-02234-4.

## Background

Catatonia is characterized by psychomotor alterations and reduced contact with the environment. The underlying pathophysiology of this condition remained unclear since its initial description by Kahlbaum in 1874 [1]. Initially linked to schizophrenia [2], it has later been found to also occur in the setting of mood disorders or various organic conditions [3]. In 2013, the Diagnostic and Statistical Manual of Mental Disorders, Fifth Edition (DSM-5) individualized catatonia as a proper entity, whose diagnosis requires the presence of three or more of the following signs: catalepsy, waxy flexibility, stupor, agitation, mutism, negativism, posturing, mannerisms, stereotypies, grimacing, echolalia, and echopraxia [4]. Hence, specific clinical scales have been developed, such as the Bush-Francis Catatonia Rating Scale and the Pediatric Catatonia Rating Scale [5, 6]. The proper assessment of catatonia remains challenging, especially in youths, who tend to exhibit specific catatonic features (e.g. acrocyanosis, incontinence) [6].

Catatonia is a severe syndrome, which dramatically increases the risk of premature death [7]. In children and youth, catatonia remains poorly delineated, but appears more common in boys, with a prevalence ranging from 0.6 to 17.8% [8–10]. A neurodevelopmental predisposition to catatonia [11, 12] stemmed from its frequent association with early-onset schizophrenia [5], and from the fact that it also occurs in patients with pervasive developmental disorder (PDD) or intellectual disability [13]. The efficacy of benzodiazepines (γ-Aminobutyric acid (GABA)-A agonists) on catatonic symptoms suggests that catatonia may be triggered by an imbalance between the GABAergic and the glutamatergic system.

Among the contributing factors, several drugs have been reported to potentially trigger catatonia, such as antipsychotics, or benzodiazepine withdrawal [14–21]. The available pediatric data on catatonia are mostly based on case-reports. To date, no systematic study has been conducted to examine the relationship between drugs and catatonia in the pediatric population. Yet, drug exposure, as well as catatonic features, may vary according with the age. Thus, as drug-induced catatonia bears many uncertainties, we aimed to characterize the various patterns of drug-induced catatonia in youths, relying on the analysis of the World Health Organization (WHO) safety database (VigiBase®, Uppsala Monitoring Centre, Sweden) [22]. Furthermore, we aimed to examine the influence of age on potential drug safety signals of catatonia among the pediatric population.Indeed, both drug exposure and catatonic features may vary according to the age.

## Material and methods

### Data source

The WHO mandates the Uppsala Monitoring Centre (UMC, Sweden) to oversee drug safety [23]. Therefore, the UMC gathers evidence regarding adverse drug reactions (ADRs), leading to the identification of drug safety signals [24]. Since 1967, VigiBase®, the WHO safety database, collects Individual Case Safety Reports originating from more than 172 national pharmacovigilance network members, as well as pharmaceutical companies. VigiBase® ensures the preservation of the anonymity of both patients and notifiers [22]. According to the French clinical research law, review from an ethics committee is not required for this type of observational study. As all data from VigiBase® were deidentified, patient informed consent was not necessary.

Each Individual Case Safety Report includes sociodemographic characteristics of the patient (age, sex), administrative information (country, reporter qualification), suspected drug (indication, start and cessation dates, dose), other drug(s) administered (suspect of interacting or concomitant administration), and characteristics of the ADR(s) (reaction(s), seriousness, onset, outcome). An ADR is defined as ‘an appreciably harmful or unpleasant reaction resulting from an intervention related to the use of a medicinal product, which usually predict a hazard for future administration and warrant prevention, or specific treatment, or alteration of the dosage regimen, or withdrawal of the product’ [25].

From a pharmacovigilance perspective, an ADR is considered to be serious if it required hospitalization or its prolongation, caused a congenital malformation, resulted in persistent or significant disability or incapacity, was life-threatening, resulted in death or needed significant medical intervention to prevent one of the abovementioned outcomes [26, 27].

### Primary analysis

#### Query

According to the Medical Dictionary for Regulatory Activities (MedDRA, version 25.1 [28]), a Preferred Term (PT) is a distinct descriptor for a symptom, sign, disease diagnosis, therapeutic indication, investigation, surgical or medical procedure, and medical social or family history characteristic.

We first queried VigiBase® for all reports featuring the PT ‘catatonia’ registered between November 14th 1967 (first reports in VigiBase®) and December 8th 2022. Then, queried reports involving patients aged under 18 years were classified into 3 groups according to their age: under 23 months (infants), 2–11 years (children), and 12–17 years (adolescents).

#### Statistical analyses

Qualitative variables were described using proportions. Quantitative variables were described in terms of means with standard deviations (±SD). Statistical analyses were performed using GraphPad Prism version 8.0.2.

Further, a disproportionality analysis was performed [29] for each drug accounting for at least 5 cases involving the ‘catatonia’ PT.

In pharmacovigilance, disproportionality analyses aim to detect and investigate potential signals regarding drug safety [29]. The superiority of the proportion of reports with a given ADR and a specific drug (cases) over the proportion of reports with the same ADR and other drugs (non-cases), allows one to suggest an association between this ADR and this drug. This disproportionality analysis was based on the assessment of both information component (IC) and reporting odds ratio (ROR). Potential drug–ADR associations were first selected by using IC_025_. Then, the ROR of each drug–ADR association allowed us to investigate the strength of previously selected signals.

The IC, which provides a comparison between observed and expected values for a drug-ADR combination, to check for a potential association, is a tool validated by UMC [22, 30]. Indeed, it favors the reduction in the risk of false-positive signals, especially if the ADR shows a low expected frequency in the database. The positivity of the IC reflects a superiority of the number of observed reports over the number of expected reports. The lower bound of the 95% confidence interval (CI) of the IC, the IC_025_, is required by the UMC to statistically confirm the detection of a signal in VigiBase® [22, 30].

As an approximate of the odds ratio (used in case-control studies), the ROR is estimated, in case–non-case studies, to assess the strength of disproportionality. An ROR equal to 1 indicates the absence of signal, revealing an equal reporting of the ADR between the drug of interest and the other drugs. Conversely, an ROR greater than 1 suggests the existence of a signal, cases being more frequently reported with the drug of interest. The higher the ROR, the stronger the association. The precision of the approximate ROR is reflected by a 95% CI. Thus, an ROR is considered to be statistically significant when the lower boundary of its 95% CI exceeds 1 [31].

Then, to be deemed as significant in this disproportionality analysis, a given drug safety signal must exhibit both a positive IC_025_ and a ROR 95% CI lower bound superior to 1.

### Secondary analyses

#### Query

To enrich our analysis, we also aimed to investigate specific types of catatonia, and catatonic features as well. Indeed, catatonia, for which diagnosis is sometimes difficult, is not always identified as such [32]. Beyond catatonia cases, we applied the same query and ranking to the following PTs:

- ‘malignant catatonia’
- ‘withdrawal catatonia’
- ‘automatism’
- ‘echolalia’
- ‘echopraxia’
- ‘posturing’
- ‘waxy flexibility’

In addition, using the initial query (Primary analysis) and striving to take into account the peculiarities of pediatric catatonia [18], we sought to distinguish the reports involving the ‘catatonia’ PT, and featuring the following PTs:

- ‘agitation’
- ‘eating disorder’
- ‘speech disorder’
- ‘stereotypy’
- ‘social avoidant behaviour’

#### Statistical analyses

Potential drug safety signals were sought for each drug accounting for at least 5 cases with the following PTs:

- ‘malignant catatonia’
- ‘withdrawal catatonia’
- ‘automatism’
- ‘echolalia’
- ‘echopraxia’
- ‘posturing’
- ‘waxy flexibility’

The analysis of catatonic features aimed to highlight specific age-dependent manifestations, and to increase the specificity of our findings.

For each age range, the complete list of active ingredients associated with catatonia, its specific types and its features mentioned above were reported in Supplementary information.

#### Healthcare professionals reporting

As a sensitivity analysis, the proportions of cases issued by healthcare professionals (physicians, pharmacists, other healthcare professionals, as defined in VigiBase®), were calculated for the following PTs:

- ‘catatonia’
- ‘automatism’
- ‘echolalia’
- ‘echopraxia’
- ‘posturing’
- ‘waxy flexibility’

## Results

### Characteristics of pediatric reports

As of December 8th 2022, the query of ‘catatonia’ in Vigibase® yielded 3897 reports. Among these, 421 (10.8%) concerned patients below 18 years of age, mainly belonging to the 12-to-17-year group (274, 65.1%), and originating from the United States (307, 72.9%). Healthcare professionals reported most of the cases (242, 57.4%). Details regarding the characteristics of the reports are available in Table 1.

**Table 1:** Characteristics of the reports of pediatric patients with catatonia

Characteristics	Number of reports (%)			
	0 days–23 months	2–11 years	12–17 years	Total < 18 years
Total	37 (100)	110 (100)	274 (100)	421 (100)
Sex				
Female	15 (40.5)	47 (42.8)	137 (50.0)	199 (47.2)
Male	21 (56.8)	63 (57.3)	136 (49.6)	220 (52.3)
Unknown	1 (2.7)		1 (0.4)	2 (0.5)
Country				
United States of America	23 (62.2)	79 (71.8)	205 (74.8)	307 (72.9)
Canada	5 (13.5)	5 (4.5)	8 (2.9)	18 (4.3)
Germany	1 (2.7)	1 (0.9)	12 (4.4)	14 (3.3)
United Kingdom	2 (5.4)	2 (1.8)	9 (3.3)	13 (3.1)
France		3 (2.7)	5 (1.8)	8 (1.9)
Italy	1 (2.7)	2 (1.8)	5 (1.8)	8 (1.9)
China	2 (5.4)	4 (3.6)		6 (1.4)
Australia	1 (2.7)	2 (1.8)	3 (1.1)	6 (1.4)
Czechia		4 (3.6)	1 (0.4)	5 (1.2)
Switzerland		1 (0.9)	3 (1.1)	4 (1.0)
Korea		1 (0.9)	2 (0.7)	3 (0.7)
Portugal			3 (1.1)	3 (0.7)
Türkiye			3 (1.1)	3 (0.7)
Netherlands	1 (2.7)	1 (0.9)	1 (0.4)	3 (0.7)
India			2 (0.7)	2 (0.5)
Poland			2 (0.7)	2 (0.5)
Sweden			2 (0.7)	2 (0.5)
Finland	1 (2.7)		1 (0.4)	2 (0.5)
Belgium			1 (0.4)	1 (0.2)
Greece			1 (0.4)	1 (0.2)
Ireland			1 (0.4)	1 (0.2)
Israel		1 (0.9)		1 (0.2)
Japan			1 (0.4)	1 (0.2)
Lithuania			1 (0.4)	1 (0.2)
Namibia			1 (0.4)	1 (0.2)
New Zealand		1 (0.9)		1 (0.2)
Norway		1 (0.9)		1 (0.2)
Oman		1 (0.9)		1 (0.2)
Romania		1 (0.9)		1 (0.2)
Spain			1 (0.4)	1 (0.2)
Reporter qualification				
Healthcare Professional	8 (21.6)	56 (50.9)	178 (65.0)	242 (57.4)
Physician	5 (13.5)	34 (30.9)	124 (45.3)	163 (38.7)
Pharmacist		5 (4.5)	4 (1.5)	9 (2.1)
Other Health Professional	3 (8.1)	17 (15.5)	50 (18.2)	70 (16.6)
Others	6 (16.2)	17 (15.5)	41 (15.0)	135 (13.2)
Lawyer			2 (0.7)	2 (0.7)
Consumer	6 (16.2)	17 (15.5)	39 (14.2)	62 (14.7)
Unknown	23 (62.2)	38 (34.5)	59 (21.5)	120 (28.5)

Risperidone (86, 20.4%), olanzapine (84, 19.9%), quetiapine (39, 9.3%), and aripiprazole (33, 7.8%) accounted for more than half (242, 57.5%) of the catatonia reports. Overall, 312 (87.4%) cases were deemed serious, among which 4 deaths (1.3%) occured. When the outcome was available (125, 29.7%), almost all (115, 92.0%) patients recovered or were recovering at the time of the analysis, 8 (6.4%) did not recover, and 2 (1.6%) recovered with sequelae. Among cases reported by healthcare professionals, risperidone (73, 30.2%) and olanzapine (68, 28.1%) were prevailing.

#### Catatonic features

Among catatonic features in the pediatric population, in terms of absolute number of cases, ‘posturing’ (194 reports) was followed by ‘echolalia’ (83), ‘automatism’ (33), ‘waxy flexibility’ (20), and ‘echopraxia’ (10). When focusing on healthcare professionals reports, 61 cases of ‘posturing’, 38 cases of ‘echolalia’, 23 cases of ‘automatism’, 13 cases of ‘waxy flexibility’ and 9 cases of ‘echopraxia’ were yielded.

### Infants (from birth to 2 years of age)

Infants accounted for 37 (8.8%) of the pediatric reports of catatonia retrieved in VigiBase®. In this age range, 21 (56.8%) patients were male, and their mean age was 6.3 (±4.6) months. Only one case involved a newborn.

The most frequently co-reported symptoms were: ‘unresponsive to stimuli’ (10, 27.0%), ‘pyrexia’ (9, 24.3%), and ‘seizure’ (8, 21.6%). In terms of proportions, vaccines led the list of catatonia-associated medicines.

In this age range, 20 (74.1%) were deemed serious. When follow-up was available (15, 40.5%), most of the infants (13, 86.7%) recovered or were recovering, 1 (6.7%) was not recovering, and 1 (6.7%) recovered with sequelae at the time of the analysis.

Regarding ‘catatonia’, in terms of disproportionality, vaccines were leading again.

#### Catatonic features

**Fig. 1:**
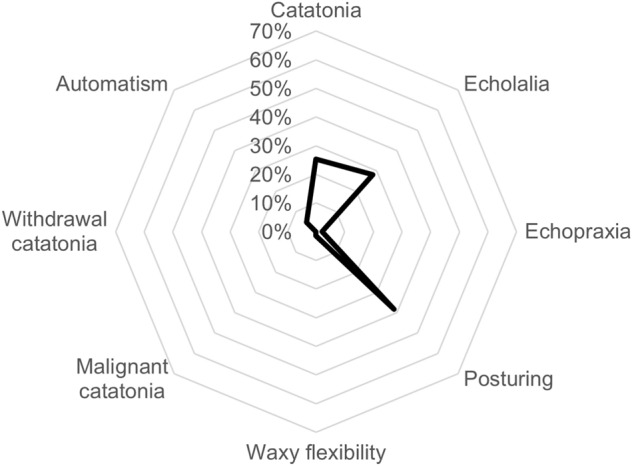
Distribution of catatonia-related preferred terms in infants

This age group included 56 ‘posturing’ cases, 41 ‘echolalia’ cases, 7 ‘automatism’ cases, 3 ‘echopraxia’ cases, and 2 ‘waxy flexibility’ cases, as displayed in Fig. 1. The complete list of active ingredients associated with catatonia and its features in infants is available in Table S1.

One case involving both ‘catatonia’ and an ‘eating disorder’ was found, ascribed to several vaccines (pneumococcal, hepatitis B, rotavirus, HIB, Polio, DTP). In addition, 3 cases involving both ‘catatonia’ and ‘speech disorder’ were yielded, 2 of them being attributed to measles, mumps, rubella (MMR) vaccine, and the remaining one to DTP vaccine.

Valproic acid (ROR 82.9; 95% CI 38.3–179.7), MMR vaccine (ROR 6.8; 95% CI 3.7–12.5), and hepatitis B vaccine (ROR 4.7; 95% CI 2.0–11.1) led to a disproportionality signal for ‘echolalia’ symptom. The three strongest signals for ‘posturing’ symptom involved vaccines. The full disproportionality analysis for cases of catatonia, echolalia and posturing in infants is available in Table 2.

**Table 2:** Disproportionality analysis for reports of catatonia, echolalia and posturing in infants

Preferred term	Active ingredient	IC_025_	ROR	95% CI	Number (%)
Catatonia	Pneumococcal vaccine	0.1	2.7	1.4–5.4	13 (35.1)
	DTP, Polio, HIb vaccine	1.2	8.0	3.8–17.0	9 (24.3)
Echolalia	MMR vaccine	1.2	6.8	3.7–12.5	18 (43.9)
	Valproic acid	2.6	82.9	38.3–179.7	8 (19.5)
	Hepatitis B vaccine	1.2	4.7	2.0–11.1	6 (14.6)
Posturing	Pneumococcal vaccine	1.1	6.2	3.7–10.6	31 (55.4)
	DTP vaccine	0.2	2.5	1.4–4.4	15 (26.8)
	Rotavirus vaccine	0.5	3.3	1.8–6.0	13 (23.2)
	VZV vaccine	0.7	4.5	2.3–8.9	10 (17.9)
	Hepatitis A vaccine	0.4	5.8	2.3–14.5	5 (8.9)
	DTP, Polio, Hepatitis B vaccine	0.6	6.9	2.8–17.3	5 (8.9)

*ROR* Reporting Odds Ratio, *IC* Information Component, *CI* Confidence Interval, *DTP* Diphtheria, Tetanus, Pertussis, *HIB* Haemophilus Influenzae type B, *MMR* Measles, Mumps, Rubella, *VZV* Varicella Zoster Virus

### Children (2–11 years)

Children accounted for 110 catatonia reports, representing 26.1% of pediatric catatonia cases of the database. The majority of children were male (63, 57.3%), with a mean age of 7.3 (±2.7) years. Seizure (15, 13.6%), agitation (11, 10.0%), and speech disorder (10, 9.1%) were the most frequently co-reported symptoms. The most reported drugs were ciclosporin (9, 8.2%), prednisolone (8, 7.3%), and risperidone (7, 6.4%).

In this age group, 67 cases (76.1%) were considered to be serious, with 1 (1.1%) fatality. Among reports with available follow-up (34, 30.9%), 32 (94.1%) children recovered or were in the process of recovering, and 2 (5.9%) were not recovering. One patient died from a neuroleptic malignant syndrome.

The three strongest signals detected for catatonia involved haloperidol (ROR 104.3; 95% CI 45.6–238.5), ondansetron (ROR 40.5; 95% CI 16.5–99.5), and ciclosporin (ROR 27.4; 95% CI 13.8–54.1).

#### Catatonic features

**Fig. 2:**
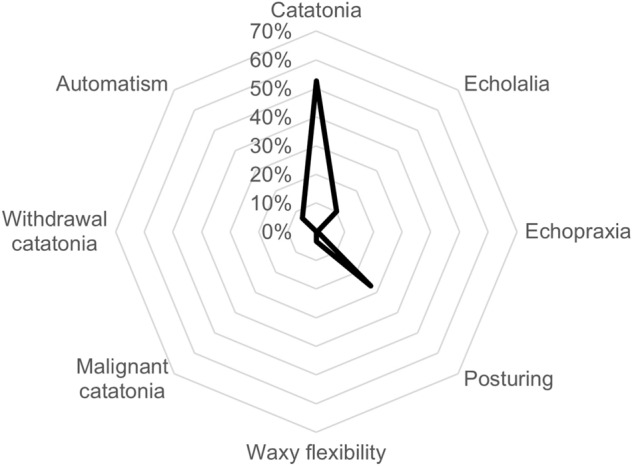
Distribution of catatonia-related preferred terms in children

Regarding catatonic features, children were involved in 56 ‘posturing’ cases, 21 ’echolalia’ cases, 14 ‘automatism’ cases, 7 ‘waxy flexibility’ cases, and 1 ‘echopraxia’ case, as shown in Fig. 2. The complete list of active ingredients involved is available in Table S2.

One case involving both ‘catatonia’ and ‘stereotypy’ was retrieved among children, and was related to ciclosporin and methylprednisolone. In addition, 10 cases involving both ‘catatonia’ and ‘speech disorder’ were identified, 2 of them being related to HPV vaccine. Then, 11 cases of ‘catatonia’ featuring ‘agitation’ were found, mainly involving methylphenidate, amifostine, and HPV vaccine (2 reports each).

All posturing disproportionality signals in children were subsequent to vaccines. The disproportionality analysis for reports of catatonia and posturing in children years is displayed in Table 3.

**Table 3:** Disproportionality analysis for reports of catatonia and posturing in children

Preferred Term	Active Ingredient	IC_025_	ROR	95% CI	Number (%)
Catatonia	Ciclosporin	2.4	27.4	13.8–54.1	9 (8.2)
	Prednisolone	2.1	23.1	11.2–47.4	8 (7.3)
	Risperidone	1.5	12.6	5.9–27.1	7 (6.4)
	Methylprednisolone	1.5	15.5	6.8–35.3	6 (5.5)
	Haloperidol	2.2	104.3	45.6–238.5	6 (5.5)
	Midazolam	1.5	26.6	10.9–65.4	5 (4.5)
	Ondansetron	1.6	40.5	16.5–99.5	5 (4.5)
Posturing	HPV vaccine	2.1	16.4	8.5–31.7	11 (19.6)
	DTP vaccine	0.3	2.9	1.5–5.7	11 (19.6)
	Meningococcal vaccine	1.1	6.1	3.1–12.1	10 (17.9)
	Influenza vaccine	0.6	4.6	2.2–9.8	8 (14.3)
	Hepatitis a vaccine	1.0	9.2	3.9–21.4	6 (10.7)

*ROR* Reporting Odds Ratio, *IC* Information Component, *CI* Confidence Interval, *DTP* Diphtheria, Tetanus, Pertussis, *HPV* Human Papillomavirus

### Adolescents (12–17 years)

Adolescents aged between 12 and 17 years accounted for 274 (65.1%) of the pediatric reports of catatonia. In this age group, 137 (50.0%) patients were female. Their mean age was 15.1 (±1.6) years, with almost one-third being at the upper range of adolescence (17-year-old accounting for 79 cases, 28.8%). The most frequently co-reported terms were ‘neuroleptic malignant syndrome’ (60, 21.9%), ‘drug ineffective’ (23, 8.4%), and ‘insomnia’ (23, 8.4%). Olanzapine accounted for 84 (30.7%) records, followed by risperidone (79, 28.8%), quetiapine (39, 14.2%), and aripiprazole (33, 12.0%). This age range included 128 (46.7%) patients presenting with a psychiatric medical history.

When available, 225 (93.0%) reports were deemed serious, including 3 (1.3%) deaths. Among reports displaying follow-up (76, 27.7%), 70 (92.1%) adolescents recovered or were recovering, 5 (6.6%) did not recovered, and 1 (1.3%) recovered with sequelae. One patient recovered from catatonia with sequelae, while 3 other patients died (from encephalitis in 2 cases, and myocarditis in another case).

Chlorpromazine (ROR 199.1; 95% CI 134.8–294.1), benzatropine (ROR 193; 95% CI 104.1–361.6), and olanzapine (ROR 135.7; 95% CI 104.6–175.9) had the greatest RORs.

#### Specific types and catatonic features

Regarding ‘malignant catatonia’, 16 reports were retrieved, mainly involving risperidone (12, 75.0%) and methylprednisolone (62.5%). No death was reported among these cases. Regarding ‘withdrawal catatonia’, our query yielded 6 reports. All of them involved olanzapine and lorazepam (6; 100%).

**Fig. 3:**
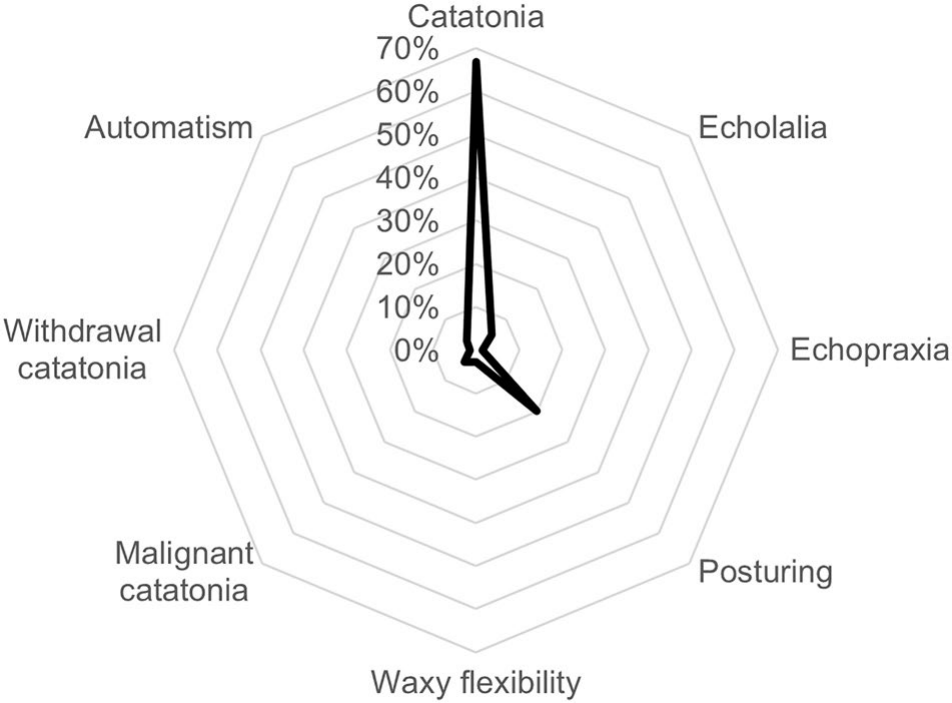
Distribution of catatonia-related preferred terms in adolescents

Regarding catatonic features, 82 ‘posturing’, 21 ‘echolalia’, 12 ‘automatism’, 11 ‘waxy flexibility’, and 6 ‘echopraxia’ reports were retrieved in VigiBase®, as shown in Fig. 3. The complete list of suspected active ingredients for this age group is available in Table S3.

One case involving both ‘catatonia’ and an ‘eating disorder’ was retrieved among adolescents, and was related to valaciclovir. In addition, 4 reports involved both ‘catatonia’ and ‘stereotypy’, among which 3 reports were linked to risperidone (75.0%), and olanzapine (75.0%). In addition, 7 cases involving both ‘catatonia’ and ‘speech disorder’ were yielded, among which 2 were attributed to yellow fever vaccine. Then, 19 reports of ‘catatonia’ featuring ‘agitation’ were retrieved, with a predominance of risperidone (9 reports), olanzapine (7 reports), and quetiapine (4 reports). ‘Social avoidant behaviour’ accounted for 9 cases of catatonia in adolescents, and mainly involved bupropion (6 reports).

Valproic acid (ROR 65.1; 95% CI 26.3–161.2) and olanzapine (ROR 148.8; 95% CI 60.0–368.9) were both subject to a disproportionality signal for ‘echolalia’. For ‘posturing’, chlorpromazine (ROR 99.8; 95% CI 40.2–247.5) was leading, followed by other antipsychotics. The overall disproportionality analysis is available in Table 4.

**Table 4:** Disproportionality analysis for reports of catatonia, echolalia, posturing, malignant and withdrawal catatonia in adolescents

Preferred term	Active ingredient	IC_025_	ROR	95% CI	Number (%)
Catatonia	Olanzapine	5.6	135.7	104.6–175.9	84 (30.7)
	Risperidone	4.4	43.9	33.8–57.1	79 (28.8)
	Quetiapine	4.3	47.5	33.8–66.8	39 (14.2)
	Aripiprazole	3.6	25.5	17.7–36.7	33 (12.0)
	Haloperidol	4.3	60.0	41.4–87.0	32 (11.7)
	Chlorpromazine	4.9	199.1	134.8–294.1	30 (10.9)
	Ziprasidone	4.5	133.7	87.9–203.4	25 (9.1)
	Lorazepam	4.2	85.1	54.7–132.3	22 (8.0)
	Clozapine	3.2	23.7	15.2–37.1	21 (7.7)
	Valproic acid	2.1	9.2	5.7–14.8	18 (6.6)
	Lithium	3.7	69.2	42.1–113.6	17 (6.2)
	Benzatropine	3.4	193.4	104.1–361.6	11 (4.0)
	Diphenhydramine	2.5	27.0	14.3–50.9	10 (3.6)
	Fluoxetine	1.5	7.9	4.2–15.0	10 (3.6)
	Sertraline	1.5	8.0	4.2–15.0	10 (3.6)
	Lamotrigine	1.0	5.9	2.9–11.9	8 (2.9)
	Bupropion	1.7	12.2	6.0–24.7	8 (2.9)
	Clonazepam	1.9	20.7	9.8–44.1	7 (2.6)
	Topiramate	1.7	14.9	7.0–31.5	7 (2.6)
	Diazepam	1.6	18.1	8.0–40.7	6 (2.2)
	Chloroquine	2.0	45.8	20.2–103.4	6 (2.2)
	Guanfacine	1.3	20.0	8.2–48.5	5 (1.8)
Echolalia	Valproic acid	2.3	65.1	26.3–161.2	7 (33.3)
	Olanzapine	2.5	148.8	60.0–368.9	7 (33.3)
Posturing	HPV vaccine	1.6	7.2	4.7–11.3	32 (39.0)
	Meningococcal vaccine	1.8	7.8	4.7–12.8	21 (25.6)
	DTP vaccine	2.3	13.6	7.7–23.7	15 (18.3)
	VZV vaccine	2.3	16.8	9.1–31.1	12 (14.6)
	Hepatitis a vaccine	2.3	19.3	10.2–36.5	11 (13.4)
	Olanzapine	2.5	36.7	18.4–73.5	9 (11.0)
	Quetiapine	1.7	22.33	9.7–51.3	6 (7.3)
	Influenza vaccine	0.7	6.2	2.7–14.1	6 (7.3)
	Risperidone	0.6	7.0	2.8–17.2	5 (6.1)
	Chlorpromazine	1.8	99.8	40.2–247.5	5 (6.1)
Malignant catatonia	Risperidone	2.5	21.5	11.6–39.9	12 (75.0)
	Methylprednisolone	2.9	62.8	32.2–122.7	10 (62.5)
Withdrawal catatonia	Olanzapine	3.1	25.1	10.9–57.9	6 (100)
	Lorazepam	3.5	77.5	33.6–178.6	6 (100)

*ROR* Reporting Odds Ratio, *IC* Information Component, *CI* Confidence Interval, *DTP* Diphtheria, Tetanus, Pertussis, *HPV* Human Papillomavirus, *VZV* Varicella Zoster Virus

## Discussion

Our analysis of the WHO safety database highlights different patterns of drug-related catatonia in infancy, childhood, and adolescence. Indeed, almost all catatonia reports in infants were attributed to vaccines. In children, both antipsychotics and non-psychotropic drugs were involved in catatonia, while vaccines still being at the forefront in cases of posturing. Last, in adolescents, catatonia was mainly ascribed to psychotropic drugs (antipsychotics, antidepressants, mood stabilizers), which potential involvement was also confirmed in the secondary analysis (malignant catatonia, echolalia, echopraxia, posturing, and waxy flexibility). Consistently with the overall distribution of the contributions to VigiBase®, most of the catatonia cases were issued by the United States of America [33].

In infants, vaccines prevailed overall, both in terms of absolute number of cases and of disproportionality. Infections are well-known factors fostering the onset of catatonia [34, 35]. Vaccines, which may induce some degree of central nervous system (CNS) inflammation, as in influenza-like symptoms [36], might have a comparable impact. Pyrexia, being the second most co-reported term, supports this hypothesis. However, only pneumococcal vaccine was disproportionately reported with both catatonia and posturing in this age group. Posturing, which is defined as the maintenance of a posture, was subject to disproportionality signals with vaccines in all age groups, as a possible result of vaccine-related reactogenicity, which often leads to asthenia [36]. The distinctive signal involving valproic acid and echolalia might originate from situations of prenatal maternal exposure. Indeed, in such cases, communication disorders were documented including perseverative activities, gesture imitation, and echolalia, as components of the fetal valproate syndrome [37, 38].

In children, various drug classes were involved in catatonia. Consistently with previous findings [14, 39, 40], ciclosporin, prednisolone and methylprednisolone stood out in terms of disproportionality. The mechanisms of these associations remain unclear, eventhough ciclosporin’s neurological central effects are known [41], and high-dose steroids commonly lead to neuropsychiatric side effects, such as hypomania, depression, argumentativeness, and behavioral abnormalities [42, 43]. Two antipsychotics were also disproportionately reported. The first is risperidone, for which the Food and Drug Administration (FDA) and the European Medicines Agency (EMA) have granted marketing authorization in the treatment of irritability in children with an autistic disorder from the age of 5 [44, 45]. The second is haloperidol, authorized for marketing by the EMA in persistent, severe aggression in children with autism or PDD [46] over the age of 6. As catatonic symptoms may be due to an antipsychotic-related reduced tolerance to stress (through a dopaminergic imbalance), patients’ psychiatric history per se might constitute a vulnerability factor to catatonia [5, 47]. A few catatonia cases related to dopamine antagonist antiemetics have been reported previously. Likewise, we singled out a drug safety signal for ondansetron, a serotonin 5-HT_3_ receptor antagonist. In fact, ondansetron is known to increase the risk of serotonin syndrome, and has also been reported in some cases of neuroleptic malignant syndrome [48, 49]. Differential diagnosis between these two entities might be challenging, as autonomic disturbances and neuromuscular hyperexcitability are prominent features of both syndromes, and polypharmacy (often including antidepressants and antipsychotic drugs) may prevent the clinician from incriminating one class or another in the first place. Several studies concur with the hypothesis that neuroleptic-induced catatonia would be a part of a continuum of autonomic and extrapyramidal alterations, ending with neuroleptic malignant syndrome [50, 51]. Indeed, considering the shared thermoregulatory dysfunction, hypermetabolic state, and abnormalities in laboratory findings, malignant catatonia and neuroleptic malignant syndrome could belong to the same broad entity [52]. Regarding catatonic features, both agitation and speech disorders accounted for about 10% of catatonia reports involving children. These findings may seem surprising, as agitated catatonia is not rare, and as schizophasia and verbigeration are common features of pediatric catatonia [18, 53]. However, these symptoms belonging to the Bush-Francis Catatonia Rating Scale and the Pediatric Catatonia Rating Scale, they were probably under-reported aside from the diagnosis of catatonia [6, 54].

Similarly, in adolescents, the query fetched a few cases of risperidone-related malignant catatonia (previously termed ‘lethal catatonia’ [55]), and neuroleptic malignant syndrome was co-reported in more than one-fifth of the cases. In addition, eight antipsychotic safety signals were in favor of neuroleptic-induced catatonia, half of them being confirmed by concomitant signals of posturing disorders. Chlorpromazine had the highest ROR in this age group, which possibly concurs with the perceived different risk of catatonia [56] between typical and atypical antipsychotics. Indeed, a ‘thalamocortical loop’ connects lateral and medial orbitofrontal cortical areas to the striatum, pallidum and substantia nigra, through the subcortex. In return, this dopaminergic circuit is modulated by GABA, and to a lesser extent by serotonergic projections issued from the dorsal raphae [47]. Aside from antipsychotics, mood stabilizers and antidepressants accounted for three safety signals, reflecting the multiple neurotransmitter imbalances that may underlie catatonia [15, 57]. In addition, we shed some light on a new signal involving benzatropine, a drug that has been reported concomitantly in a neuroleptic malignant syndrome case [58]. As an anticholinergic drug, prescribed to correct antipsychotic-induced motor impairments, it came as no surprise that benzatropine induced dysautonomic and gesture alterations in a predisposed population [59], and constituted another factor of CNS disequilibrium in adjunction to antipsychotics.

While other studies reported suspected drug-induced catatonia cases [14–17], our study is, to our knowledge, the first comprehensive pediatric pharmacovigilance analysis. It highlighted different susceptibility profiles, depending on the age of the patients. In addition, using terms related to catatonic features, it included a secondary analysis to increase the specificity of our findings.

Though, this study is hindered by the inherent flaws of spontaneous reporting systems and post-marketing pharmacovigilance approaches, such as under-reporting [60]. Indeed, the incompleteness of data did not allow us to assess the characteristics of sequelae in patients who had not fully recovered at the time of the analysis. The lack of follow-up may have, somehow, increased the number of cases classified as ‘not recovered’. The diagnosis of catatonia might have been unclear for some notifiers, either because of young patients’ age or due to potential overlap of symptoms with other diseases. This may have led to a substantial coding heterogeneity. Likewise, the assessment of catatonic symptomatology via clinical scales (i.e. Pediatric Catatonia Rating Scale [6]) could not be verified because of the relative scarcity of information contained in some reports. A major limitation is that the distinction between cases involving validated diagnoses of catatonia and cases involving catatonia-like symptomatology cannot be ensured, and this distinction relies on the prior analysis of the notifier and the pharmacovigilance expert who registered the case in the database. Furthermore, patients’ medical history could have constituted a confounding factor in the assessment of the causality. Yet, these reporting biases may, at least partly, have been mitigated by the fact that most of the reporters were healthcare professionals. Indeed, the dominance (in terms of absolute number of cases) of risperidone and olanzapine was confirmed in our focus on healthcare professionals’ notifications. Moreover, the diagnosis of catatonia being particularly difficult, even for clinicians, reports issued by non-healthcare professionals may have relied on a prior medical assessment. In that context, the inclusion of cases reported by consumers in our disproportionality analyses was intended to make up for the paucity of pediatric reports in the database. As VigiBase® does not include data regarding illegal substances, their potential role in the onset of catatonia cannot be taken into account. Last, pharmacovigilance studies are exploratory, characterizing potential drug safety signals, rather than concluding on the causality. Population-based studies should help in reassessing these outcomes.

In this study, based on a comprehensive analysis of catatonia reports from the WHO pharmacovigilance database, we confirmed the overall importance of neuroleptic-induced catatonia in pediatric population. We characterized three safety profiles, according to the patients’ age ranges, with specific semiological patterns. While catatonia was mainly attributed to vaccines in infants, it was ascribed to ciclosporin, steroids and antipsychotics in children, and most likely involved psychotropic drugs in adolescents. We also highlighted the role of less suspected drugs, such as ondansetron.

In case of newly emerged psychiatric and motor symptoms in young patients, a careful anamnesis may allow one to disentangle a possible side effect from medical history and acute illnesses. Given the diversity of catatonic features, and the fact that catatonia often leads to serious comorbidities, early suspicion of drug involvement may help to guide the diagnosis and provide appropriate care. When an overlap with neuroleptic malignant syndrome exists, an antipsychotic involvement should also promptly be evoked, as this condition may be life-threatening.

### Supplementary Information

Below is the link to the electronic supplementary material.

Supplementary file1 (DOCX 22 KB)

Supplementary file2 (DOCX 25 KB)

Supplementary file3 (DOCX 28 KB)

## Data Availability

The data that support the findings of this study are available from Uppsala Monitoring Center (UMC) but restrictions apply to the availability of these data, which were used under license for the current study, and so are not publicly available. Access to VigiBase® is available without fees to Dr Fanny Rocher. Data are however available from the authors upon reasonable request and with permission of UMC.
