# Supplementary material for: Drug-related catatonia in youths: real-world insights from the WHO Safety Database
Source: Eur Child Adolesc Psychiatry. 2023 Jun 12;33(5):1383–93. doi: 10.1007/s00787-023-02234-4 (PMC11098911; doi:10.1007/s00787-023-02234-4)
Supplement: Supplementary file 1 — Supplementary file1 (DOCX 22 KB) [file 787_2023_2234_MOESM1_ESM.docx]

European Child and Adolescent Psychiatry

Drug-related Catatonia in Youths: Real-World Insights from the WHO Safety Database

Diane Merino ^a b^ (ORCID : 0000-0001-7669-2339), Alexandre O. Gérard ^b^ (ORCID : 0000-0001-6591-6966), Thibaud Lavrut ^b^, Florence Askenazy ^c d^ (ORCID : 0000-0002-3821-0965), Susanne Thümmler ^c d^ (ORCID 0000-0001-9993-6981), François Montastruc ^e #^ (ORCID: 0000-0001-7056-8126), Milou-Daniel Drici ^b #^ (ORCID: 0000-0003-4121-530X)

^a^ Department of Psychiatry, University Hospital of Nice, Nice, France

^b^ Department of Pharmacology and Pharmacovigilance Center of Nice, University Hospital Center of Nice, Nice, France

^c^ Department of Child and Adolescent Psychiatry, Children’s Hospitals of Nice, CHU-Lenval Nice, France

^d^ CoBTek Laboratory, Université Côte d’Azur, 06000 Nice, France

^e^ Department of Medical and Clinical Pharmacology, Centre of PharmacoVigilance and Pharmacoepidemiology, Faculty of Medicine, Toulouse University Hospital, Toulouse, France

^#^ The two authors contributed equally to this work as the last authors

**Correspondence to:**

Milou-Daniel DRICI

Department of Pharmacology and Pharmacovigilance,

Côte d’Azur University

Pasteur Hospital, Bât J4,

30 Avenue de la Voie Romaine - CS51069,

06001 Nice Cedex 01, France

Email: pharmacovigilance@chu-nice.fr

Tel = +33 492 034 708

Fax = +33 492 034 709

**Table S1. Reported suspected drugs in infants with catatonia, echolalia, echopraxia, posturing, waxy flexibility or automatism**

| **Preferred Term** | **Active Ingredient** | **Number (%)** |
| --- | --- | --- |
| **Catatonia** | Pneumococcal vaccine | 13 (35.1) |
|  | DTP, Polio, HIB vaccine | 9 (24.3) |
|  | HIB vaccine | 7 (20.0) |
|  | DTP vaccine | 7 (20.0) |
|  | MMR vaccine | 5 (13.5) |
|  | Hepatitis b vaccine | 3 (8.1) |
|  | Polio vaccine | 3 (8.1) |
|  | Meningococcal vaccine | 2 (5.4) |
|  | Varicella zoster vaccine | 2 (5.4) |
|  | Paracetamol | 1 (2.7) |
|  | Flupentixol | 1 (2.7) |
|  | Deptropine | 1 (2.7) |
|  | Fluoxetine | 1 (2.7) |
|  | Ganciclovir | 1 (2.7) |
|  | Paroxetine | 1 (2.7) |
|  | Vigabatrin | 1 (2.7) |
|  | Cisapride | 1 (2.7) |
|  | Rotavirus vaccine | 1 (2.7) |
|  | Palivizumab | 1 (2.7) |
|  | Interferon alfa-2b | 1 (2.7) |
|  | Ganglioside : gm1 | 1 (2.7) |
|  | DTP, Polio, Hepatitis b vaccine | 1 (2.7) |
|  | DTP, Hepatitis b vaccine | 1 (2.7) |
|  | Quetiapine | 1 (2.7) |
| **Echolalia** | MMR vaccine | 18 (43.9) |
|  | HIB vaccine | 9 (22.0) |
|  | Valproic acid | 8 (19.5) |
|  | DTP vaccine | 6 (14.6) |
|  | Hepatitis b vaccine | 6 (14.6) |
|  | Polio vaccine | 3 (7.3) |
|  | Pneumococcal vaccine | 2 (4.9) |
|  | VZV vaccine | 2 (4.9) |
|  | Hepatitis a vaccine | 1 (2.4) |
|  | DTP, Polio, HIB, Hepatitis b vaccine | 1 (2.4) |
|  | MMRV vaccine | 1 (2.4) |
|  | DTP, Polio vaccine | 1 (2.4) |
| **Echopraxia** | Palivizumab | 1 (33.3) |
|  | Pneumococcal vaccine | 1 (33.3) |
|  | Valproic acid | 1 (33.3) |
| **Posturing** | Pneumococcal vaccine | 31 (55.4) |
|  | DTP vaccine | 15 (26.8) |
|  | Rotavirus vaccine | 13 (23.2) |
|  | HIB vaccine | 11 (19.6) |
|  | Polio vaccine | 10 (17.9) |
|  | VZV vaccine | 10 (17.9) |
|  | MMR vaccine | 10 (17.9) |
|  | DTP, Polio, HIB vaccine | 6 (10.7) |
|  | Hepatitis a vaccine | 5 (8.9) |
|  | DTP, Polio, Hepatitis b vaccine | 5 (8.9) |
|  | Hydrocortisone | 4 (7.1) |
|  | Methotrexate | 4 (7.1) |
|  | Daunorubicin | 4 (7.1) |
|  | Asparaginase | 4 (7.1) |
|  | Cytarabine | 4 (7.1) |
|  | Etoposide | 4 (7.1) |
|  | Hepatitis b vaccine | 4 (7.1) |
|  | Fludarabine | 4 (7.1) |
|  | Hepatitis b, HIB vaccine | 4 (7.1) |
|  | Morphine | 3 (5.4) |
|  | Influenza vaccine | 3 (5.4) |
|  | Naloxone | 2 (3.6) |
|  | Rabies vaccine | 1 (1.8) |
|  | Fentanyl | 1 (1.8) |
|  | Flibanserin | 1 (1.8) |
|  | DTP, Polio, HIB, Hepatitis b vaccine | 1 (1.8) |
|  | MMRV vaccine | 1 (1.8) |
|  | Baclofen | 1 (1.8) |
|  | Methylergotamine | 1 (1.8) |
| **Waxy flexibility** | Atropine | 1 (50.0) |
|  | Pneumococcal vaccine | 1 (50.0) |
| **Automatism** | Pneumococcal vaccine | 3 (42.8) |
|  | MMR vaccine | 1 (14.3) |
|  | Hepatitis a vaccine | 1 (14.3) |
|  | Rotavirus vaccine | 1 (14.3) |
|  | Polio vaccine | 1 (14.3) |
|  | DTP vaccine | 1 (14.3) |
|  | DTP, Polio, HIB, Hepatitis b vaccine | 1 (14.3) |
|  | DTP, HIB, Hepatitis b vaccine | 1 (14.3) |
|  | DTP, Polio vaccine | 1 (14.3) |

DTP: Diphtheria, Tetanus, Pertussis; HIB: Haemophilus Influenzae type B; HPV: Human Papillomavirus; MMR: Measles, Mumps, Rubella; MMRV: Measles, Mumps, Rubella, Varicella VZV: Varicella Zoster Virus
