# Supplementary material for: Drug-related catatonia in youths: real-world insights from the WHO Safety Database
Source: Eur Child Adolesc Psychiatry. 2023 Jun 12;33(5):1383–93. doi: 10.1007/s00787-023-02234-4 (PMC11098911; doi:10.1007/s00787-023-02234-4)
Supplement: Supplementary file 2 — Supplementary file2 (DOCX 25 KB) [file 787_2023_2234_MOESM2_ESM.docx]

European Child and Adolescent Psychiatry

Drug-related Catatonia in Youths: Real-World Insights from the WHO Safety Database

Diane Merino ^a b^ (ORCID : 0000-0001-7669-2339), Alexandre O. Gérard ^b^ (ORCID : 0000-0001-6591-6966), Thibaud Lavrut ^b^, Florence Askenazy ^c d^ (ORCID : 0000-0002-3821-0965), Susanne Thümmler ^c d^ (ORCID 0000-0001-9993-6981), François Montastruc ^e #^ (ORCID: 0000-0001-7056-8126), Milou-Daniel Drici ^b #^ (ORCID: 0000-0003-4121-530X)

^a^ Department of Psychiatry, University Hospital of Nice, Nice, France

^b^ Department of Pharmacology and Pharmacovigilance Center of Nice, University Hospital Center of Nice, Nice, France

^c^ Department of Child and Adolescent Psychiatry, Children’s Hospitals of Nice, CHU-Lenval Nice, France

^d^ CoBTek Laboratory, Université Côte d’Azur, 06000 Nice, France

^e^ Department of Medical and Clinical Pharmacology, Centre of PharmacoVigilance and Pharmacoepidemiology, Faculty of Medicine, Toulouse University Hospital, Toulouse, France

^#^ The two authors contributed equally to this work as the last authors

**Correspondence to:**

Milou-Daniel DRICI

Department of Pharmacology and Pharmacovigilance,

Côte d’Azur University

Pasteur Hospital, Bât J4,

30 Avenue de la Voie Romaine - CS51069,

06001 Nice Cedex 01, France

Email: pharmacovigilance@chu-nice.fr

Tel = +33 492 034 708

Fax = +33 492 034 709

**Table S2. Reported suspected drugs in children with catatonia, echolalia, echopraxia, posturing, waxy flexibility or automatism**

| **Preferred Term** | **Active Ingredient** | **Number (%)** |
| --- | --- | --- |
| **Catatonia** | Ciclosporin | 9 (8.2) |
|  | Prednisolone | 8 (7.3) |
|  | Risperidone | 7 (6.4) |
|  | Methylprednisolone | 6 (5.5) |
|  | Haloperidol | 6 (5.5) |
|  | Midazolam | 5 (4.5) |
|  | Ondansetron | 5 (4.5) |
|  | Methylphenidate | 4 (3.6) |
|  | Sertraline | 4 (3.6) |
|  | Influenza vaccine | 4 (3.6) |
|  | DTP vaccine | 4 (3.6) |
|  | Lorazepam | 3 (2.7) |
|  | Prochlorperazine | 3 (2.7) |
|  | Montelukast | 3 (2.7) |
|  | Atomoxetine | 3 (2.7) |
|  | Polio vaccine | 3 (2.7) |
|  | Guanfacine | 2 (1.8) |
|  | Propofol | 2 (1.8) |
|  | Aripiprazole | 2 (1.8) |
|  | Diphenhydramine | 2 (1.8) |
|  | Ethosuximide | 2 (1.8) |
|  | Promethazine | 2 (1.8) |
|  | Metoclopramide | 2 (1.8) |
|  | Pneumococcal vaccine | 2 (1.8) |
|  | Somatropin | 2 (1.8) |
|  | Hepatitis b vaccine | 2 (1.8) |
|  | Amifostine | 2 (1.8) |
|  | Oseltamivir | 2 (1.8) |
|  | Influenza A(H1N1)pdm09 vaccine | 2 (1.8) |
|  | VZV vaccine | 2 (1.8) |
|  | HPV vaccine | 2 (1.8) |
|  | Diphtheria, Tetanus vaccine | 2 (1.8) |
|  | Covid-19 vaccine | 2 (1.8) |
|  | Lisinopril | 1 (0.9) |
|  | Amlodipine | 1 (0.9) |
|  | Ketamine | 1 (0.9) |
|  | Chlorpromazine | 1 (0.9) |
|  | Ibuprofen | 1 (0.9) |
|  | Cisplatin | 1 (0.9) |
|  | Clobazam | 1 (0.9) |
|  | Topiramate | 1 (0.9) |
|  | Macrogol 3350 | 1 (0.9) |
|  | Metronidazole | 1 (0.9) |
|  | Erythromycin | 1 (0.9) |
|  | Mannitol | 1 (0.9) |
|  | Sultiame | 1 (0.9) |
|  | Prednisone | 1 (0.9) |
|  | Methotrexate | 1 (0.9) |
|  | Clonazepam | 1 (0.9) |
|  | Clozapine | 1 (0.9) |
|  | Oxatomide | 1 (0.9) |
|  | Mercurous chloride | 1 (0.9) |
|  | Oxybate sodium | 1 (0.9) |
|  | Chlorproethazine | 1 (0.9) |
|  | Lamotrigine | 1 (0.9) |
|  | Hepatitis a vaccine | 1 (0.9) |
|  | Mirtazapine | 1 (0.9) |
|  | Levetiracetam | 1 (0.9) |
|  | Voriconazole | 1 (0.9) |
|  | Dexmethylphenidate | 1 (0.9) |
|  | Paliperidone | 1 (0.9) |
|  | HIB vaccine | 1 (0.9) |
|  | Meningococcal vaccine | 1 (0.9) |
|  | Loratadine; Pseudoephedrine | 1 (0.9) |
|  | DTP, HIB vaccine | 1 (0.9) |
|  | MMR vaccine | 1 (0.9) |
|  | DTP, Polio vaccine | 1 (0.9) |
| **Echolalia** | Valproic acid | 3 (14.3) |
|  | MMR vaccine | 3 (14.3) |
|  | Risperidone | 2 (9.5) |
|  | DTP vaccine | 1 (4.8) |
|  | Chloroquine | 1 (4.8) |
|  | Bcg vaccine | 1 (4.8) |
|  | Prednisolone | 1 (4.8) |
|  | Haloperidol | 1 (4.8) |
|  | Methylprednisolone | 1 (4.8) |
|  | Methylphenidate | 1 (4.8) |
|  | Clonidine | 1 (4.8) |
|  | Lorazepam | 1 (4.8) |
|  | Pneumococcal vaccine | 1 (4.8) |
|  | Ciclosporin | 1 (4.8) |
|  | Budesonide | 1 (4.8) |
|  | Hepatitis b vaccine | 1 (4.8) |
|  | Azithromycin | 1 (4.8) |
|  | Sertraline | 1 (4.8) |
|  | Lansoprazole | 1 (4.8) |
|  | Topiramate | 1 (4.8) |
|  | Rituximab | 1 (4.8) |
|  | Immunoglobulin g human | 1 (4.8) |
|  | Atomoxetine | 1 (4.8) |
|  | Polio vaccine | 1 (4.8) |
|  | Meningococcal vaccine | 1 (4.8) |
|  | Varicella zoster vaccine | 1 (4.8) |
|  | Amoxicillin;Clavulanic acid | 1 (4.8) |
|  | DTP, Polio, HIB, Hepatitis b vaccine | 1 (4.8) |
| **Echopraxia** | Valproic acid | 1 (100) |
| **Posturing** | HPV vaccine | 11 (19.6) |
|  | DTP vaccine | 11 (19.6) |
|  | Meningococcal vaccine | 10 (17.9) |
|  | Influenza vaccine | 8 (14.3) |
|  | Hepatitis a vaccine | 6 (10.7) |
|  | Baclofen | 4 (7.1) |
|  | VZV vaccine | 4 (7.1) |
|  | Methotrexate | 3 (5.4) |
|  | MMR vaccine | 3 (5.4) |
|  | Paracetamol | 2 (3.6) |
|  | Lansoprazole | 2 (3.6) |
|  | Trihexyphenidyl | 2 (3.6) |
|  | Hydrocortisone | 2 (3.6) |
|  | Methylphenidate | 2 (3.6) |
|  | Diazepam | 2 (3.6) |
|  | Lidocaine | 2 (3.6) |
|  | Mercaptopurine | 2 (3.6) |
|  | Magnesium hydroxide | 2 (3.6) |
|  | Vancomycin | 2 (3.6) |
|  | Aripiprazole | 2 (3.6) |
|  | Influenza A(H1N1)pdm09 vaccine | 2 (3.6) |
|  | Polio vaccine | 2 (3.6) |
|  | Vincristine | 1 (1.8) |
|  | Morphine | 1 (1.8) |
|  | Dexamethasone | 1 (1.8) |
|  | Ceftriaxone | 1 (1.8) |
|  | Risperidone | 1 (1.8) |
|  | Pegaspargase | 1 (1.8) |
|  | Haloperidol | 1 (1.8) |
|  | Doxycycline | 1 (1.8) |
|  | Cytarabine | 1 (1.8) |
|  | Doxorubicin | 1 (1.8) |
|  | Anti-d immunoglobulin | 1 (1.8) |
|  | Vigabatrin | 1 (1.8) |
|  | Cetirizine | 1 (1.8) |
|  | Bosentan | 1 (1.8) |
|  | Escitalopram | 1 (1.8) |
|  | Blinatumomab | 1 (1.8) |
|  | Measles vaccine | 1 (1.8) |
|  | Covid-19 vaccine | 1 (1.8) |
| **Waxy flexibility** | Flupentixol | 2 (28.6) |
|  | Influenza A(H1N1)pdm09 vaccine | 2 (28.6) |
|  | Valproic acid | 1 (14.3) |
|  | Tramadol | 1 (14.3) |
|  | Miglustat | 1 (14.3) |
| **Automatism** | Fentanyl | 3 (21.4) |
|  | Midazolam | 3 (21.4) |
|  | Dimenhydrinate | 2 (14.3) |
|  | Morphine | 2 (14.3) |
|  | Lisdexamfetamine | 2 (14.3) |
|  | MMR vaccine | 2 (14.3) |
|  | Chloral hydrate | 1 (7.1) |
|  | Dexamethasone | 1 (7.1) |
|  | Diazepam | 1 (7.1) |
|  | Cyclophosphamide | 1 (7.1) |
|  | Hydrocortisone | 1 (7.1) |
|  | Prednisone | 1 (7.1) |
|  | Hydroxyzine | 1 (7.1) |
|  | Vincristine | 1 (7.1) |
|  | Methylphenidate | 1 (7.1) |
|  | Mercaptopurine | 1 (7.1) |
|  | Methotrexate | 1 (7.1) |
|  | Asparaginase | 1 (7.1) |
|  | Cytarabine | 1 (7.1) |
|  | Glycerol | 1 (7.1) |
|  | Folinic acid | 1 (7.1) |
|  | Propofol | 1 (7.1) |
|  | Influenza A(H1N1)pdm09 vaccine | 1 (7.1) |
|  | Meningococcal vaccine | 1 (7.1) |
|  | DTP vaccine | 1 (7.1) |
|  | COVID-19 vaccine | 1 (7.1) |

DTP: Diphtheria, Tetanus, Pertussis; HIB: Haemophilus Influenzae type B; HPV: Human Papillomavirus; MMR: Measles, Mumps, Rubella; MMRV: Measles, Mumps, Rubella, Varicella VZV: Varicella Zoster Virus
