# Supplementary material for: Drug-related catatonia in youths: real-world insights from the WHO Safety Database
Source: Eur Child Adolesc Psychiatry. 2023 Jun 12;33(5):1383–93. doi: 10.1007/s00787-023-02234-4 (PMC11098911; doi:10.1007/s00787-023-02234-4)
Supplement: Supplementary file 3 — Supplementary file3 (DOCX 28 KB) [file 787_2023_2234_MOESM3_ESM.docx]

European Child and Adolescent Psychiatry

Drug-related Catatonia in Youths: Real-World Insights from the WHO Safety Database

Diane Merino ^a b^ (ORCID : 0000-0001-7669-2339), Alexandre O. Gérard ^b^ (ORCID : 0000-0001-6591-6966), Thibaud Lavrut ^b^, Florence Askenazy ^c d^ (ORCID : 0000-0002-3821-0965), Susanne Thümmler ^c d^ (ORCID 0000-0001-9993-6981), François Montastruc ^e #^ (ORCID: 0000-0001-7056-8126), Milou-Daniel Drici ^b #^ (ORCID: 0000-0003-4121-530X)

^a^ Department of Psychiatry, University Hospital of Nice, Nice, France

^b^ Department of Pharmacology and Pharmacovigilance Center of Nice, University Hospital Center of Nice, Nice, France

^c^ Department of Child and Adolescent Psychiatry, Children’s Hospitals of Nice, CHU-Lenval Nice, France

^d^ CoBTek Laboratory, Université Côte d’Azur, 06000 Nice, France

^e^ Department of Medical and Clinical Pharmacology, Centre of PharmacoVigilance and Pharmacoepidemiology, Faculty of Medicine, Toulouse University Hospital, Toulouse, France

^#^ The two authors contributed equally to this work as the last authors

**Correspondence to:**

Milou-Daniel DRICI

Department of Pharmacology and Pharmacovigilance,

Côte d’Azur University

Pasteur Hospital, Bât J4,

30 Avenue de la Voie Romaine - CS51069,

06001 Nice Cedex 01, France

Email: pharmacovigilance@chu-nice.fr

Tel = +33 492 034 708

Fax = +33 492 034 709

**Table S3. Reported suspected drugs in adolescents with catatonia, echolalia, echopraxia, posturing, waxy flexibility, automatism, withdrawal or malignant catatonia**

| **Preferred Term** | **Active Ingredient** | **Number (%)** |
| --- | --- | --- |
| **Catatonia** | Olanzapine | 84 (30.7) |
|  | Risperidone | 79 (28.8) |
|  | Quetiapine | 39 (14.2) |
|  | Aripiprazole | 33 (12.0) |
|  | Haloperidol | 32 (11.7) |
|  | Chlorpromazine | 30 (10.9) |
|  | Ziprasidone | 25 (9.1) |
|  | Lorazepam | 22 (8.0) |
|  | Clozapine | 21 (7.7) |
|  | HPV vaccine | 21 (7.7) |
|  | Valproic acid | 18 (6.6) |
|  | Lithium | 17 (6.2) |
|  | Benzatropine | 11 (4.0) |
|  | Diphenhydramine | 10 (3.6) |
|  | Fluoxetine | 10 (3.6) |
|  | Sertraline | 10 (3.6) |
|  | Lamotrigine | 8 (2.9) |
|  | Bupropion | 8 (2.9) |
|  | Clonazepam | 7 (2.6) |
|  | Topiramate | 7 (2.6) |
|  | Meningococcal vaccine | 7 (2.6) |
|  | Diazepam | 6 (2.2) |
|  | Methylphenidate | 6 (2.2) |
|  | Atomoxetine | 6 (2.2) |
|  | Chloroquine | 6 (2.2) |
|  | Guanfacine | 5 (1.8) |
|  | Covid-19 vaccine | 5 (1.8) |
|  | Clonidine | 4 (1.5) |
|  | Lurasidone | 4 (1.5) |
|  | Phenobarbital | 4 (1.5) |
|  | Phenytoin | 4 (1.5) |
|  | Methotrexate | 3 (1.1) |
|  | Midazolam | 3 (1.1) |
|  | Varicella zoster vaccine | 3 (1.1) |
|  | Escitalopram | 2 (0.7) |
|  | Metoprolol | 2 (0.7) |
|  | Citalopram | 2 (0.7) |
|  | Alprazolam | 2 (0.7) |
|  | Biperiden | 2 (0.7) |
|  | Fluvoxamine | 2 (0.7) |
|  | Furosemide | 2 (0.7) |
|  | Droperidol | 2 (0.7) |
|  | Yellow fever vaccine | 2 (0.7) |
|  | Tetrabenazine | 2 (0.7) |
|  | Loxapine | 2 (0.7) |
|  | Tiapride | 2 (0.7) |
|  | Cyamemazine | 2 (0.7) |
|  | Zuclopenthixol | 2 (0.7) |
|  | Gabapentin | 2 (0.7) |
|  | Hepatitis a vaccine | 2 (0.7) |
|  | Lansoprazole | 2 (0.7) |
|  | Felbamate | 2 (0.7) |
|  | Valaciclovir | 2 (0.7) |
|  | Influenza vaccine | 2 (0.7) |
|  | Oseltamivir | 2 (0.7) |
|  | Sirolimus | 2 (0.7) |
|  | Paliperidone | 2 (0.7) |
|  | Hepatitis a vaccine; Hepatitis b vaccine | 2 (0.7) |
|  | Elexacaftor;Ivacaftor;Tezacaftor | 2 (0.7) |
|  | Hydrocortisone | 1 (0.4) |
|  | Benztropeine | 1 (0.4) |
|  | Prednisone | 1 (0.4) |
|  | Sulfamethoxazole;Trimethoprim | 1 (0.4) |
|  | Tetracycline | 1 (0.4) |
|  | Cyclophosphamide | 1 (0.4) |
|  | Promethazine | 1 (0.4) |
|  | Carbamazepine | 1 (0.4) |
|  | Hydroxyzine | 1 (0.4) |
|  | Pimozide | 1 (0.4) |
|  | Oxcarbazepine | 1 (0.4) |
|  | Ethyl loflazepate | 1 (0.4) |
|  | Mefloquine | 1 (0.4) |
|  | Chorionic gonadotrophin | 1 (0.4) |
|  | Montelukast | 1 (0.4) |
|  | Levetiracetam | 1 (0.4) |
|  | Fluphenazine | 1 (0.4) |
|  | Trihexyphenidyl | 1 (0.4) |
|  | Atropine | 1 (0.4) |
|  | Amobarbital | 1 (0.4) |
|  | Mepivacaine | 1 (0.4) |
|  | Chlorprothixene | 1 (0.4) |
|  | Prochlorperazine | 1 (0.4) |
|  | Prednisolone | 1 (0.4) |
|  | Pethidine | 1 (0.4) |
|  | Dexamfetamine | 1 (0.4) |
|  | Immunoglobulin human normal | 1 (0.4) |
|  | Propranolol | 1 (0.4) |
|  | Levomepromazine | 1 (0.4) |
|  | Benzonatate | 1 (0.4) |
|  | Prilocaine | 1 (0.4) |
|  | Bromocriptine | 1 (0.4) |
|  | Flurbiprofen | 1 (0.4) |
|  | Flunarizine | 1 (0.4) |
|  | Ciclosporin | 1 (0.4) |
|  | Isotretinoin | 1 (0.4) |
|  | Oxybate sodium | 1 (0.4) |
|  | Propofol | 1 (0.4) |
|  | Naltrexone | 1 (0.4) |
|  | Buspirone | 1 (0.4) |
|  | Zolpidem | 1 (0.4) |
|  | Zonisamide | 1 (0.4) |
|  | Hypericum perforatum | 1 (0.4) |
|  | Rivastigmine | 1 (0.4) |
|  | Levosalbutamol | 1 (0.4) |
|  | Adalimumab | 1 (0.4) |
|  | Dexmedetomidine | 1 (0.4) |
|  | Influenza A(H1N1)pdm09 vaccine | 1 (0.4) |
|  | Corticorelin (human) | 1 (0.4) |
|  | Dextromethorphan;Paracetamol;Phenylephrine | 1 (0.4) |
|  | DTP vaccine | 1 (0.4) |
|  | DTP, Polio vaccine | 1 (0.4) |
|  | ACTH | 1 (0.4) |
| **Echolalia** | Valproic acid | 7 (33.3) |
|  | Olanzapine | 7 (33.3) |
|  | Risperidone | 4 (19.0) |
|  | Quetiapine | 3 (14.3) |
|  | Lorazepam | 2 (9.5) |
|  | Haloperidol | 2 (9.5) |
|  | Aripiprazole | 2 (9.5) |
|  | Chlorpromazine | 2 (9.5) |
|  | Lithium | 2 (9.5) |
|  | Methotrexate | 2 (9.5) |
|  | Clozapine | 2 (9.5) |
|  | Sertraline | 2 (9.5) |
|  | Topiramate | 2 (9.5) |
|  | Levetiracetam | 1 (4.8) |
|  | Ziprasidone | 1 (4.8) |
|  | Trihexyphenidyl | 1 (4.8) |
|  | Amfetamine | 1 (4.8) |
|  | Benzatropine | 1 (4.8) |
|  | Dexamfetamine | 1 (4.8) |
|  | Phenytoin | 1 (4.8) |
|  | Phenobarbital | 1 (4.8) |
|  | Immunoglobulin human normal | 1 (4.8) |
|  | Carbamazepine | 1 (4.8) |
|  | Methylphenidate | 1 (4.8) |
|  | Clonidine | 1 (4.8) |
|  | Cisplatin | 1 (4.8) |
|  | Guanfacine | 1 (4.8) |
|  | Oxcarbazepine | 1 (4.8) |
|  | Fluvoxamine | 1 (4.8) |
|  | Naltrexone | 1 (4.8) |
|  | Fluoxetine | 1 (4.8) |
|  | Buspirone | 1 (4.8) |
|  | Amisulpride | 1 (4.8) |
|  | Benztropeine | 1 (4.8) |
|  | Lamotrigine | 1 (4.8) |
|  | Atomoxetine | 1 (4.8) |
|  | HPV vaccine | 1 (4.8) |
|  | Dextromethorphan;Paracetamol;Phenylephrine | 1 (4.8) |
|  | Nitrous oxide;Oxygen | 1 (4.8) |
| **Echopraxia** | Olanzapine | 5 (83.3) |
|  | Lorazepam | 2 (33.3) |
|  | Risperidone | 2 (33.3) |
|  | Quetiapine | 2 (33.3) |
|  | Aripiprazole | 2 (33.3) |
|  | Clonidine | 1 (16.7) |
|  | Trihexyphenidyl | 1 (16.7) |
|  | Benzatropine | 1 (16.7) |
|  | Dexamfetamine | 1 (16.7) |
|  | Haloperidol | 1 (16.7) |
|  | Lithium | 1 (16.7) |
|  | Cyproheptadine | 1 (16.7) |
|  | Methylphenidate | 1 (16.7) |
|  | Methotrexate | 1 (16.7) |
|  | Valproic acid | 1 (16.7) |
|  | Oxcarbazepine | 1 (16.7) |
|  | Naltrexone | 1 (16.7) |
|  | Fluoxetine | 1 (16.7) |
|  | Buspirone | 1 (16.7) |
|  | Ondansetron | 1 (16.7) |
|  | Sertraline | 1 (16.7) |
|  | Atomoxetine | 1 (16.7) |
| **Posturing** | HPV vaccine | 32 (39.0) |
|  | Meningococcal vaccine | 21 (25.6) |
|  | DTP vaccine | 15 (18.3) |
|  | VZV vaccine | 12 (14.6) |
|  | Hepatitis a vaccine | 11 (13.4) |
|  | Olanzapine | 9 (11.0) |
|  | Quetiapine | 6 (7.3) |
|  | Influenza vaccine | 6 (7.3) |
|  | Risperidone | 5 (6.1) |
|  | Chlorpromazine | 5 (6.1) |
|  | Baclofen | 4 (4.9) |
|  | Bupropion | 4 (4.9) |
|  | Ziprasidone | 3 (3.7) |
|  | Valproic acid | 3 (3.7) |
|  | Aripiprazole | 3 (3.7) |
|  | Covid-19 vaccine | 3 (3.7) |
|  | Lorazepam | 2 (2.4) |
|  | Lithium | 2 (2.4) |
|  | Paracetamol | 2 (2.4) |
|  | Haloperidol | 2 (2.4) |
|  | Hepatitis b vaccine | 2 (2.4) |
|  | Lamotrigine | 2 (2.4) |
|  | Atomoxetine | 2 (2.4) |
|  | Polio vaccine | 2 (2.4) |
|  | Vincristine | 1 (1.2) |
|  | Prednisone | 1 (1.2) |
|  | Methylphenidate | 1 (1.2) |
|  | Methotrexate | 1 (1.2) |
|  | Clonidine | 1 (1.2) |
|  | Clozapine | 1 (1.2) |
|  | Guanfacine | 1 (1.2) |
|  | Fluoxetine | 1 (1.2) |
|  | Ethanol | 1 (1.2) |
|  | Trihexyphenidyl | 1 (1.2) |
|  | Benzatropine | 1 (1.2) |
|  | Dexamfetamine | 1 (1.2) |
|  | Phenytoin | 1 (1.2) |
|  | Cyclophosphamide | 1 (1.2) |
|  | Phenobarbital | 1 (1.2) |
|  | Morphine | 1 (1.2) |
|  | Amphotericin b | 1 (1.2) |
|  | Methadone | 1 (1.2) |
|  | Yellow fever vaccine | 1 (1.2) |
|  | Asparaginase | 1 (1.2) |
|  | Cocaine | 1 (1.2) |
|  | Diamorphine | 1 (1.2) |
|  | Cannabis sativa | 1 (1.2) |
|  | Ifosfamide | 1 (1.2) |
|  | Bupivacaine | 1 (1.2) |
|  | Cyclobenzaprine | 1 (1.2) |
|  | Oxcarbazepine | 1 (1.2) |
|  | Naltrexone | 1 (1.2) |
|  | Buspirone | 1 (1.2) |
|  | Paroxetine | 1 (1.2) |
|  | Ondansetron | 1 (1.2) |
|  | Benztropeine | 1 (1.2) |
|  | Sertraline | 1 (1.2) |
|  | Topiramate | 1 (1.2) |
|  | Venlafaxine | 1 (1.2) |
|  | Imiglucerase | 1 (1.2) |
|  | Miglustat | 1 (1.2) |
|  | Dextromethorphan;Paracetamol;Phenylephrine | 1 (1.2) |
|  | MMR vaccine | 1 (1.2) |
|  | Diphtheria;Tetanus vaccine | 1 (1.2) |
| **Waxy flexibility** | Olanzapine | 4 (36.4) |
|  | Haloperidol | 3 (27.3) |
|  | Chlorpromazine | 2 (18.2) |
|  | Levomepromazine | 2 (18.2) |
|  | Imipramine | 2 (18.2) |
|  | Sulpiride | 2 (18.2) |
|  | Risperidone | 2 (18.2) |
|  | Valproic acid | 1 (9.1) |
|  | Trihexyphenidyl | 1 (9.1) |
|  | Lithium | 1 (9.1) |
|  | Lorazepam | 1 (9.1) |
|  | Clonazepam | 1 (9.1) |
|  | Bromocriptine | 1 (9.1) |
|  | Sertraline | 1 (9.1) |
|  | Quetiapine | 1 (9.1) |
|  | Aripiprazole | 1 (9.1) |
|  | Influenza A(H1N1)pdm09 vaccine | 1 (9.1) |
|  | HPV vaccine | 1 (9.1) |
| **Malignant**  **catatonia** | Risperidone | 12 (75.0) |
|  | Methylprednisolone | 10 (62.5) |
|  | Haloperidol | 4 (25.0) |
|  | Ziprasidone | 3 (18.8) |
|  | Clonidine | 2 (12.5) |
|  | Clozapine | 2 (12.5) |
|  | Quetiapine | 2 (12.5) |
|  | Olanzapine | 2 (12.5) |
|  | Aripiprazole | 2 (12.5) |
|  | Levetiracetam | 1 (6.3) |
|  | Chlorpromazine | 1 (6.3) |
|  | Rituximab | 1 (6.3) |
| **Automatism** | Influenza A(H1N1)pdm09 vaccine | 4 (33.3) |
|  | Isotretinoin | 2 (16.7) |
|  | Olanzapine | 2 (16.7) |
|  | Methotrexate | 1 (8.3) |
|  | Valproic acid | 1 (8.3) |
|  | Triazolam | 1 (8.3) |
|  | Clobazam | 1 (8.3) |
|  | Alizapride | 1 (8.3) |
|  | Lamotrigine | 1 (8.3) |
|  | Topiramate | 1 (8.3) |
|  | Miglustat | 1 (8.3) |
| **Withdrawal catatonia** | Olanzapine | 6 (100) |
|  | Lorazepam | 6 (100) |

DTP: Diphtheria, Tetanus, Pertussis; HIB: Haemophilus Influenzae type B; HPV: Human Papillomavirus; MMR: Measles, Mumps, Rubella; MMRV: Measles, Mumps, Rubella, Varicella VZV: Varicella Zoster Virus
